# CDKN3 in cancer: context-dependent cell-cycle control and implications for genome stability and therapy

**DOI:** 10.3389/fonc.2026.1991125

**Published:** 2026-10-30

**Authors:** Yi Li, Long Cheng, Xiaowei Zhang, Zhenkun Zhang, Guobo Du

**Affiliations:** 1 Department of Oncology, Affiliated Hospital of North Sichuan Medical College, Nanchong, Sichuan, China; 2 Key Laboratory of Integrated Precision Diagnosis and Treatment for Solid Tumors, Sichuan Provincial Health Commission, Nanchong, Sichuan, China; 3 Department of Pain, People’s Hospital of Yilong County, Nanchong, Sichuan, China

**Keywords:** CDKN3, cell-cycle control, chromosomal instability, genome stability, hepatocellular carcinoma, KAP, replication stress, therapeutic vulnerability

## Abstract

Cyclin-dependent kinase inhibitor 3 (CDKN3), also known as kinase-associated phosphatase (KAP), dephosphorylates activating cyclin-dependent kinase 2 (CDK2) Thr160, yet high expression is associated with aggressive features in some cancers. This review distinguishes messenger RNA (mRNA) expression, protein abundance, phosphatase activity, functional dependency, prognostic association, and treatment-response prediction. Renal studies include selected perturbation evidence, whereas lung studies cited here primarily report prognostic or computational associations. Hepatocellular carcinoma (HCC) findings remain directionally discordant across analytes, etiologies, models, and endpoints. Recent triple-negative breast cancer (TNBC) studies include transcriptomic and cell-perturbation evidence, as well as a separate cell and murine mammary fat-pad graft study, while an ultraviolet (UV)-induced skin-cancer study identifies a sex- and context-specific protective axis. These results do not support a universal CDKN3 role. A CDKN3-specific connection to chromosomal instability (CIN) and cyclic GMP–AMP synthase (cGAS)–stimulator of interferon genes (STING) signaling remains unvalidated. Micronuclei and CIN do not invariably activate cGAS–STING. Bulk immune signatures and survival associations alone cannot establish direct immune regulation, functional dependency, or treatment sensitivity. This focused narrative review compares molecular measurements, genetic perturbations, clinical associations, and selected computational studies. We outline matched assays, genetic rescue, longitudinal CIN measurements, and pathway-specific tests needed to determine whether CDKN3 is a context-specific dependency, a biomarker, or a therapeutic target.

## Introduction

Cyclin-dependent kinase inhibitor 3 (CDKN3), also known as kinase-associated phosphatase (KAP), is a dual-specificity phosphatase that dephosphorylates activating cyclin-dependent kinase 2 (CDK2) Thr160 in biochemical assays (1, 2). CDKN3 is also implicated in mitotic control through cyclin-dependent kinase 1 (CDK1; historically termed CDC2) Thr161 regulation (3). Yet high CDKN3 expression is frequently associated with proliferative and adverse clinical features in selected cancers (4–6). This descriptive tension is the CDKN3 paradox. The effect of inhibiting one CDK substrate cannot by itself predict a tumor’s behavior, because expression, activity, cell-cycle timing, and lineage vary.

We distinguish CDKN3 messenger RNA (mRNA) expression, protein abundance, phosphatase activity, functional phenotype, functional dependency, prognostic association, and treatment-response prediction. More transcript or protein does not necessarily mean greater catalytic activity or a tumor-cell dependency. An association with survival does not establish independent prognostic utility, differential treatment benefit, or therapeutic tractability. We use “paradox” descriptively to denote the apparent discordance between inhibitory substrate chemistry and associations between high expression and aggressive tumor features; it does not imply a demonstrated molecular switch between tumor-suppressive and oncogenic states.

This separation also defines the meaning of negative evidence. A failed RNA association in one cohort does not falsify a protein-dependent effect in another, and a knockdown phenotype does not establish an enzymatic mechanism without restoration by a properly expressed rescue construct. Similarly, an unfavorable survival association may arise because CDKN3 follows cell-cycle activity rather than because the phosphatase independently determines patient outcome.

This narrative review compares biochemical, functional, clinical, and computational evidence. We conducted the final search on 30 September 2026 in PubMed and public resources including The Cancer Genome Atlas (TCGA), Gene Expression Omnibus (GEO), and the Human Protein Atlas, using combinations of CDKN3, KAP, cancer, CDK2, hepatocellular carcinoma, chromosomal instability, micronuclei, cyclic GMP–AMP synthase (cGAS)–stimulator of interferon genes (STING), and treatment response. Titles, abstracts, and full texts were screened for peer-reviewed primary studies; conference abstracts, preprints, duplicate datasets, and reports without a CDKN3-specific measurement, perturbation, or clinical endpoint were excluded. Multigene signatures were retained as association evidence and were not interpreted as CDKN3-specific functional or predictive evidence. This was not a systematic review: no protocol registration, formal risk-of-bias assessment, or meta-analysis was performed. **Figure 1** separates the established CDK2 substrate reaction and distinct tumor-evidence streams from parallel, testable readouts proposed after CDKN3 perturbation; it does not imply a sequential mechanism.

**Figure 1:**
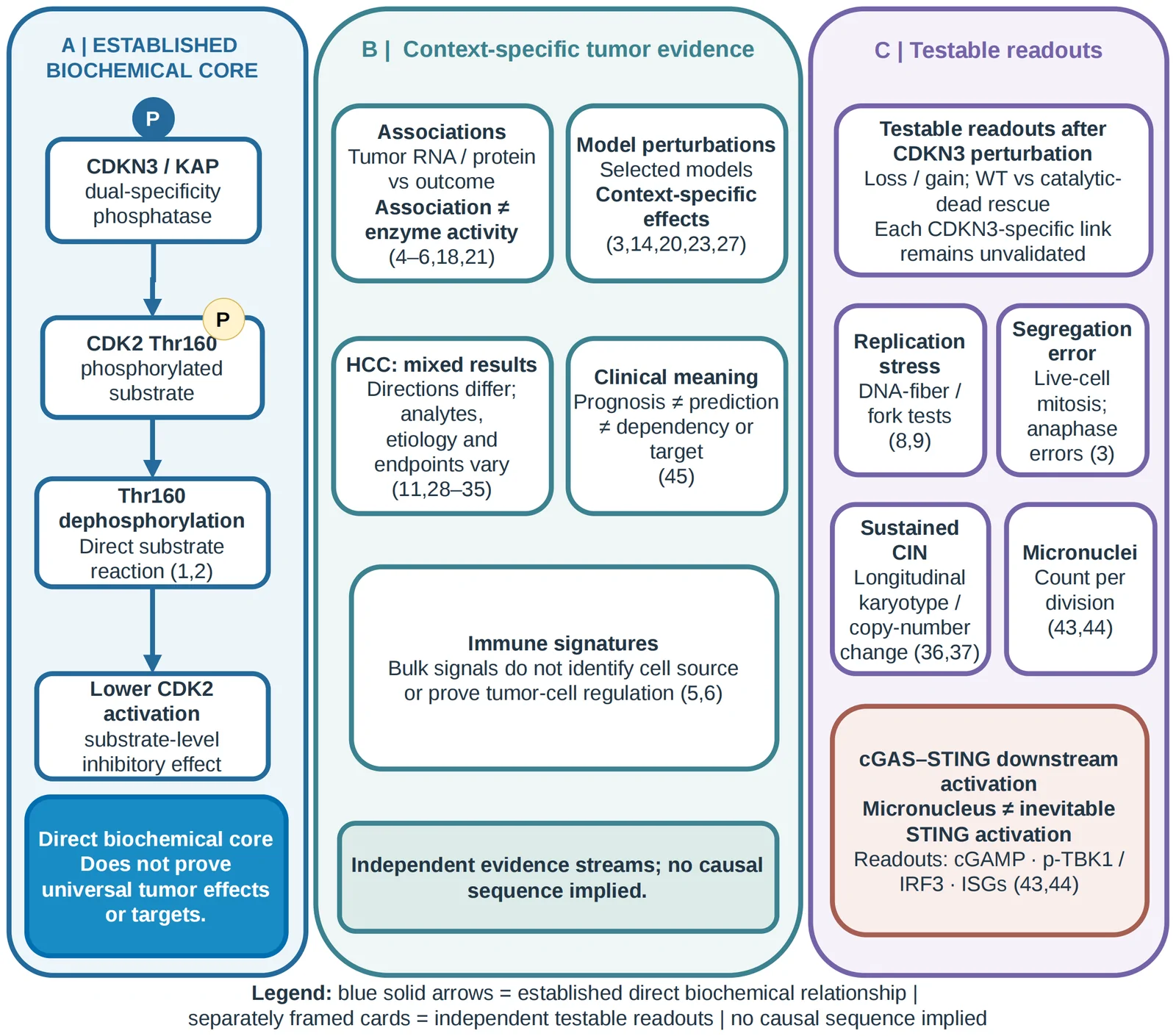
Evidence framework for CDKN3 in cancer. **(A)** Shows the established biochemical reaction: CDKN3/KAP dephosphorylates CDK2 Thr160 (1, 2). The blue panel and solid blue arrows denote this direct biochemical relationship; the teal cards in Panel B represent separate evidence streams, and the purple and red-brown cards in **(C)** denote proposed readouts rather than established CDKN3-specific effects. **(B)** separates expression–outcome associations, context-specific model perturbations, discordant hepatocellular carcinoma (HCC) findings, clinical interpretation, and bulk immune signatures. The HCC card displays selected citations (11, 28–35); HCC references 12 and 13 are additionally summarized in the main text and **Table 1**. The model evidence in reference 3 includes CDKN3-loss-associated changes in CDK1 Thr161 phosphorylation and mitotic/segregation phenotypes; these findings motivate testing the separately framed readouts in **(C)** but do not establish a route to sustained chromosomal instability (CIN). **(C)** Presents parallel, testable readouts after CDKN3 perturbation; no sequential relationship among them is implied. These readouts are replication stress (8, 9), chromosome-segregation errors (3), sustained CIN (36, 37), micronucleus frequency (43, 44), and potential downstream cGAS–STING activation (38, 39, 43). Only solid blue arrows are drawn; no dashed or gray causal connectors are shown. The blue P badge marks the phosphatase, the gold P marks phosphate on CDK2, and ≠ indicates that micronucleus formation does not necessarily mean STING activation. Micronucleus formation does not inevitably activate cGAS–STING; assess cyclic GMP–AMP (cGAMP), phosphorylated TANK-binding kinase 1 (p-TBK1), phosphorylated interferon regulatory factor 3 (p-IRF3), and interferon-stimulated genes (ISGs) (43, 44). DNA, deoxyribonucleic acid; RNA, ribonucleic acid; KAP, kinase-associated phosphatase; CDK2, cyclin-dependent kinase 2; CDK1, cyclin-dependent kinase 1; cGAS, cyclic GMP–AMP synthase; STING, stimulator of interferon genes; WT, wild type.

## Biological basis and distinct molecular layers

### Substrate chemistry and cell-cycle phenotypes

The direct biochemical anchor is CDKN3-mediated removal of phosphate from CDK2 Thr160, an activating residue (1, 2). In the assay context this is inhibitory, but its net effect on proliferation in intact cells depends on when and where the enzyme acts. CDKN3 depletion has also produced mitotic defects, altered CDK1 Thr161 phosphorylation, and chromosome-segregation abnormalities in experimental systems (3). Those findings support a role in mitotic fidelity in the studied models; they do not establish that every tumor depends on CDKN3 or that all observed mitotic effects arise from direct CDK1 dephosphorylation.

The historical name “inhibitor” can obscure this distinction. Phosphatase action on isolated CDK2 does not predict the integrated behavior of a cycling tumor cell with feedback loops, changing substrate availability, and regulated mitotic exit. The Nalepa mitotic study shows why inhibitory substrate chemistry cannot be equated with a globally growth-suppressive phenotype; the same study also does not establish a uniform pro-growth role (3). A tumor-specific perturbation must establish whether CDKN3 loss changes proliferation, survival, division fidelity, or several of these outcomes, and at what point in the cell cycle the change occurs.

A plausible bridge to genome stability is indirect. CDK2-dependent replication control suggests that changes in CDKN3 could alter replication timing or stress, while the mitotic results suggest potential effects on segregation (3, 7–9). Replication-fork stress, gap 2/mitosis (G2/M) progression, spindle defects, anaphase bridges, micronuclei, and copy-number change are therefore outcomes to measure after perturbation, not established CDKN3 downstream effects. Catalytic and noncatalytic contributions should be separated with wild-type and catalytically impaired rescue constructs.

### Transcript, protein, and activity

Cancer-associated CDKN3 transcript variants complicate the interpretation of total RNA assays (10, 11). Cell-cycle and MYC-associated transcriptional programs have been reported in hepatocellular carcinoma (HCC), while noncoding RNA and protein-interaction studies in other tumors suggest additional regulatory layers (12–17). These studies do not supply interchangeable measures of functional phosphatase activity. Isoform-specific RNA, validated protein assays, localization, CDK substrate phosphorylation, and enzyme activity should ideally be assessed in the same specimens. A high RNA signal may reflect the fraction of cycling cells; altered protein turnover or localization could produce a different functional state (4, 10).

Measurement differences also affect clinical comparisons. Bulk tumor RNA includes malignant and nonmalignant cells, whereas immunohistochemistry may depend on epitope recognition, scoring thresholds, and sampling within heterogeneous tissue. A phosphatase activity assay addresses yet another quantity and needs substrate specificity and assay controls. Studies using different layers are therefore complementary rather than direct replications. In HCC, a useful paired analysis would measure major CDKN3 isoforms, total and localized protein, and CDK2 Thr160 phosphorylation in the same tumor and adjacent tissue, recording cirrhosis, viral infection, alcohol exposure, prior treatment, and the proportion of malignant cells. Such an analysis could reveal where apparent contradictions arise without presuming a functional switch.

## Tumor-specific evidence

### Expression often accompanies proliferation and aggressive features

In lung adenocarcinoma, major CDKN3 transcripts have been associated with shorter survival, while computational analyses report CDKN3 in prognostic or immune-related signatures (4, 18, 19). These studies do not establish a CDKN3-specific perturbation phenotype or functional dependency. Renal-cell studies of circSDHC–miR-127-3p–CDKN3–E2F1 and ZNF677-associated CDKN3 regulation include functional perturbation and support a tumor-cell contribution in selected models (14, 20). An independent clinical expression association in clear-cell renal cancer remains a different level of evidence (21).

The strength of tumor-specific evidence also varies within a lineage. A survival curve derived from a clinical transcript cohort can generate a hypothesis but cannot identify whether CDKN3 itself is required by cancer cells. Gene knockdown provides a stronger test of cellular function, although off-target effects and changes in global proliferation require independent reagents and rescue. An upstream noncoding-RNA intervention is informative about a regulatory network but may affect additional genes. Even convergent studies may differ in histological subtype, treatment history, and culture conditions. These distinctions explain why the table reports evidence type beside its supported conclusion instead of assigning one unqualified role to each cancer.

In TNBC, a multi-transcriptomic study reported CDKN3 upregulation in basal subtypes and cell-line knockdown effects on G2/M progression (22). A separate study reported effects of CDKN3 manipulation on cell growth, migration/invasion, and ferroptosis-related measures, with concordant results in a 4T1 mammary fat-pad graft model (23). These model-based phenotypes do not establish clinical dependency or treatment prediction. Reports in gastric, endometrial, and pancreatic cancers describe clinical associations, cell-cycle signatures, or experimental phenotypes of differing strength (15–17, 24–26). Pan-cancer expression and survival analyses add breadth but are sensitive to proliferative state, tumor purity, and confounding (5, 6). In female ultraviolet (UV)-induced skin cancer, an estrogen receptor/E2F transcription factor 1 (E2F1)/CDKN3 axis was reported to protect against squamous cell carcinoma in the tested context (27), further cautioning against assigning CDKN3 a uniform tumor role. In neuroblastoma, CDKN3 silencing has been examined in relation to cell differentiation, whereas in low-grade glioma, high CDKN3 expression has been associated with poor prognosis (48, 49).

### Hepatocellular carcinoma shows unresolved directional discordance

HCC provides the clearest conflict and requires study-level comparison. Yeh and colleagues described aberrant KAP/CDKN3 transcripts (11). Mori et al. studied CDKN3 regulation in HepG2 cells, and Li et al. reported MYC-associated expression with model validation (12, 13). Xing and colleagues reported elevated expression and growth-associated experimental results (28); Lin and colleagues examined alcohol-related HCC and xenograft growth (29). In contrast, Dai et al. (30) reported lower protein relative to comparators, an inverse association with pathological stage, and greater survival after small interfering RNA (siRNA)-mediated CDKN3 reduction in a particular HCC cell model (30). Dai et al. (31) associated expression with outcome and tested adriamycin sensitivity after knockdown *in vitro* (31). Zhang et al. included CDKN3 among hub genes in hepatitis C virus (HCV)-associated HCC datasets (32), while Kim et al. identified it in a bulk-expression hub module from hepatitis B virus (HBV)-associated HCC (33). Dai et al. (34) included CDKN3 with a negative coefficient in a six-gene risk model from The Cancer Genome Atlas (TCGA) with Gene Expression Omnibus (GEO) evaluation (34); Wu et al. included it in a nine-gene TCGA-derived prognostic signature (35). Studies 32–35 are bulk multigene analyses, not CDKN3-specific perturbations.

The direction of these findings changes across analyte, tumor etiology, tissue comparator, antibody or transcript definition, model, intervention, and endpoint. In particular, alcohol-related, viral-hepatitis-associated, and incompletely annotated cohorts should not be pooled as though they describe the same setting. **Supplementary Table S1** compares the pivotal HCC studies by analyte, etiology, model, perturbation, endpoint, supported claim, and limitation; **Table 1** summarizes their evidence level. Together they establish unresolved directional discordance, not a confirmed oncogenic-to-tumor-suppressive switch. Matched isoform RNA, protein, catalytic readouts, and comparable perturbations in several etiology-defined models are needed before assigning a causal HCC-specific role.

**Table 1:** Representative tumor-specific evidence for CDKN3 and the conclusions most clearly supported by the current literature.

Tumor/context	Study design/evidence level	Molecular layer	Perturbation or association	Claim supported	Claim not supported/key limitation
CDKN3 biochemistry	Biochemical mechanism	Protein/activity	Substrate assays	CDKN3/KAP directly dephosphorylates CDK2 Thr160 (1, 2).	Does not establish cellular effects, tumor phosphatase activity, or therapeutic value.
Mitotic control	Cellular phenotype	Protein/mitosis	CDKN3 loss	Loss alters CDK1 Thr161 phosphorylation and chromosome-segregation phenotypes in the tested model (3).	Does not establish direct CDK1 dephosphorylation, lineage generality, or a sustained CIN phenotype.
Lung adenocarcinoma	Prognostic/computational association	RNA/signatures/cohorts	Observational and computational analyses; no gene-specific perturbation in the cited lung studies	High expression is associated with shorter survival; computational analyses include CDKN3 in prognostic or immune-related signatures (4, 18, 19).	No CDKN3-specific perturbation/rescue evidence or treatment-by-biomarker interaction in the cited studies.
Renal cell carcinoma	Regulatory-axis perturbations plus cohort association	RNA/protein/cell models	circSDHC–miR-127-3p–CDKN3–E2F1 and ZNF677-associated perturbations; separate clear-cell cohort analysis	Selected models support effects within regulatory networks; one cohort reports an expression–outcome association (14, 20, 21).	The interventions affect upstream regulators and other targets; the cohort analysis is observational and does not establish CDKN3 dependency or treatment prediction.
Hepatocellular carcinoma	Mixed tissue, cell-model, and computational evidence	RNA/protein/regulatory assays/treatment response	Expression-regulator studies (12, 13), overexpression/suppression models (28–31), and bulk signatures (32–35)	Results differ across analyte, etiology, model, treatment, and endpoint (11–13, 28–35).	Non-equivalent assays and etiologies; multigene signatures (32–35) do not isolate CDKN3 effects or establish a predictive biomarker.
TNBC: transcriptomic and cell-line study	Computational plus cellular perturbation	RNA/cell-cycle phenotype	Multi-transcriptomic analyses and CDKN3 knockdown in cells (22)	Reports basal-subtype expression patterns and a cell-line G2/M phenotype.	No xenograft or patient-level treatment-prediction evidence in this study.
TNBC: cell and graft-model study	Cellular and *in vivo* model phenotype	Protein/cells/ferroptosis	CDKN3 overexpression/knockdown; HSP90 and ferroptosis modulation; 4T1 mammary fat-pad graft (23)	Reports growth and ferroptosis-related phenotypes in cells and a 4T1 graft model.	One graft model; no patient treatment-interaction analysis or established therapeutic window.
Gastric cancer	Cell-model phenotype plus expression cohort	RNA/protein/cells	Expression analysis and cell-model perturbation, by study (24, 25)	One report describes migration/invasion and apoptosis phenotypes in cells; another evaluates expression in a clinical cohort (24, 25).	Cell assays do not establish patient-level dependency; cohort expression does not establish causality or treatment prediction.
Endometrial cancer	Cell-cycle/EMT model evidence and computational association	RNA/cells/signatures	circ-NAB1/CDKN3 cell-cycle and EMT analyses; pancancer analysis with endometrial validation (15, 26)	Reports context-specific expression and cell-cycle/EMT-related findings (15, 26).	No independent clinical treatment-prediction validation; evidence types differ between the two studies.
Pancreatic cancer	Protein-interaction and cellular phenotype	Protein/interaction/cells	GID2–CDKN3 and PSMD12–CDKN3 interaction studies (16, 17)	Reports interactions and selected growth/apoptosis phenotypes in the studied models (16, 17).	Does not establish a CDKN3 catalytic mechanism, clinical dependency, or patient treatment benefit.
Bladder cancer	Treatment-response model evidence	Protein/drug response	CDKN3 perturbation during cisplatin response (46)	Reports a CDKN3-linked cisplatin-response phenotype in the studied cells.	No treatment-by-biomarker interaction or selective therapeutic window.
Colorectal cancer	Treatment-response model evidence	Protein/drug response	CDKN3 perturbation and TIPE1-related cisplatin response (47)	Reports a model-specific association between CDKN3, TIPE1, and cisplatin response.	Cell-model response does not establish patient prediction or a treatment interaction.
Neuroblastoma	Cellular differentiation phenotype	RNA/cells	CDKN3 silencing (48)	Examines differentiation-associated phenotypes after silencing in neuroblastoma models.	Model-based evidence does not establish patient-level prognosis or dependency across tumors.
Low-grade glioma	Prognostic association	RNA/cohort	Observational expression analysis (49)	High expression is associated with poor prognosis in the analyzed cohort.	Association does not establish independent clinical utility, causality, or treatment prediction.
UV-induced skin SCC	Context-specific cellular phenotype	E2F/CDKN3/cells	UV/estrogen-receptor context and CDKN3 depletion (27)	Reports a female- and UV-context-specific protective axis in tested models.	Sex- and UV-specific model evidence; no clinical skin-cancer biomarker established.
Pan-cancer/bulk RNA	Computational association	Bulk RNA/signatures	No gene-specific perturbation in cited analyses (5, 6)	High expression co-occurs with proliferation and adverse outcome features in selected cohorts.	Tumor purity, proliferation, and cell composition confound inference; no gene-specific dependency or treatment prediction.

CDKN3, cyclin-dependent kinase inhibitor 3; KAP, kinase-associated phosphatase; CDK, cyclin-dependent kinase; HCC, hepatocellular carcinoma; HCV, hepatitis C virus; HBV, hepatitis B virus; LUAD, lung adenocarcinoma; RCC, renal cell carcinoma; TNBC, triple-negative breast cancer; HSP90, heat shock protein 90; UV, ultraviolet; SCC, squamous cell carcinoma; E2F, E2F transcription factor; CIN, chromosomal instability; RNA, ribonucleic acid; EMT, epithelial–mesenchymal transition. Evidence categories are descriptive and do not represent a quantitative quality score or interchangeable forms of evidence.

Several comparisons are particularly informative. The early transcript-variant report does not measure the same quantity as a later protein-scoring study (11, 30). Growth in a xenograft after intervention is a functional endpoint, but its model and etiology may differ from cohorts used for prognostic RNA signatures (29, 32–35). Adriamycin sensitivity is a treatment-conditioned cell phenotype rather than evidence that baseline expression predicts clinical benefit (31). The opposing results should therefore be displayed side by side, with the direction and endpoint attached to each study. Replication with the same analyte and intervention across etiologically stratified models, including orthogonal perturbation and rescue, would establish whether biological divergence persists after technical sources of disagreement are reduced.

## Genome instability and immune state

### A possible connection to chromosomal instability

Chromosomal instability (CIN) can arise through replication stress and chromosome segregation errors, and its fitness effects depend on context (36, 37). CDKN3 loss alters mitosis in defined non-HCC models (3), but a CDKN3-specific route to sustained CIN in cancer has not been demonstrated. Current evidence does not establish whether CDKN3 perturbation alters CIN, permits tolerance of pre-existing CIN, or merely co-varies with proliferative state. Time-resolved segregation and copy-number readouts with genetic rescue would be more informative than expression correlations alone.

Even if CDKN3 manipulation increased micronucleus counts, that observation alone would be insufficient to show sustained CIN. Micronuclei are one possible readout of chromosome segregation defects, whereas ongoing CIN involves repeated missegregation across cell generations. Fixed-cell counts should be paired with live-cell imaging, longitudinal karyotyping or copy-number analysis, and a quantitative comparison of divisions with and without CDKN3 manipulation. Because cell death or slowed division can shift the measured fraction of abnormal cells, investigators should report division rates and survival alongside genome-instability measurements. A rescue that reverses both the molecular change and the chromosomal phenotype would narrow the causal interpretation.

### cGAS–STING and immune-associated signatures

In some settings, cytosolic DNA generated during genome instability can engage the cyclic GMP–AMP synthase (cGAS)–stimulator of interferon genes (STING) pathway, while chronic inflammatory signaling can have tumor-supportive effects (38–42). This general biology is separate from a CDKN3-specific mechanism. Micronucleus formation, cytosolic DNA, or cGAS recruitment alone is insufficient to establish productive cGAS–STING signaling; activation depends on micronuclear envelope integrity, DNA accessibility, cGAS recruitment, cGAMP production, STING trafficking, and downstream TANK-binding kinase 1 (TBK1)–interferon regulatory factor 3 (IRF3) signaling (43, 44). Thus neither CIN nor micronucleus formation guarantees STING activation, and CDKN3 has not been established upstream of the full sequence.

The immune interpretation has an additional compartment problem. A tumor specimen contains malignant cells, fibroblasts, endothelial cells, and infiltrating leukocytes, each contributing to bulk expression profiles. An apparent relationship between CDKN3 and a computationally estimated immune population may be produced by their shared relationship with grade, purity, proliferation, stromal composition, or immune-cell abundance. Bulk immune estimates cannot determine which compartment expresses CDKN3 or establish direct immune regulation. Cell-resolved expression and spatial measurements could establish the expressing compartment, while perturbations confined to malignant cells could test whether tumor-cell signaling alters immune recruitment or function.

Studies reporting CDKN3 associations with estimated immune infiltration or checkpoint-related expression usually analyze bulk tumor transcriptomes (5, 6, 18, 21). These signatures cannot distinguish tumor-cell-intrinsic CDKN3 effects from differences in purity, proliferation, stromal composition, or immune-cell abundance. A proposed CDKN3–CIN–cGAS–STING connection should therefore be tested through CDKN3 perturbation, direct genome-instability and cytosolic-DNA measurements, cGAS localization and downstream signaling, and pathway-specific rescue or inhibition in immune-relevant models. **Figure 1** presents these as parallel testable readouts; their CDKN3-specific relationship remains unvalidated.

## Clinical significance and therapeutic vulnerabilities

Prognostic association means that a measurement correlates with outcome; independent prognostic utility requires adjustment for stage, grade, proliferation markers, and other relevant factors in a defined cohort (45). A predictive biomarker requires evidence that treatment benefit differs by biomarker status, ideally a prespecified treatment-by-marker interaction. A functional dependency requires selective fitness loss following gene-specific perturbation and rescue. Therapeutic targeting adds a further requirement for a suitable intervention, target engagement, pharmacological confirmation, and an acceptable safety window. Existing CDKN3 studies span these levels but do not collapse them into a single clinical claim.

For a prognostic claim, validation should include an assay with reproducible scoring, a prespecified cutoff, appropriate adjustment, and an independent cohort. For prediction, the design should compare treatment effects across marker-defined groups rather than correlate expression with response within treated cases. For a target claim, genetic dependency should be linked to a specific molecular mechanism and then reproduced with a tractable pharmacological intervention. Clear intended use would prevent a CDKN3 association from being interpreted as prognosis, treatment selection, and druggability (45).

Treatment resistance and therapeutic vulnerability are also distinct. CDKN3-linked cisplatin response in bladder and colorectal cell models, and adriamycin response in HCC models, are consistent with treatment-response phenotypes in the specific models studied (31, 46, 47). They do not establish differential patient benefit, a treatment-by-biomarker interaction, or selective toxicity after CDKN3 inhibition. A convincing vulnerability would require multiple gene-specific perturbations, wild-type versus catalytic-mutant rescue, relevant normal-cell comparisons, and pharmacological confirmation. CDK or DNA-damage-response inhibitors and treatment combinations are candidate strategies to test only after their mechanism and context are defined; high CDKN3 expression alone does not imply sensitivity to any such agent.

Resistance experiments are especially sensitive to the chosen dose and time point. A knockdown that changes cell survival during cisplatin treatment may also change the baseline growth rate, creating an apparent difference in drug response without a selective drug interaction. A factorial design crossing CDKN3 perturbation with drug treatment, with matched time points and baseline-growth measurements, could separate these possibilities. The same principle applies to adriamycin experiments in HCC. A clinical treatment-predictive claim would need a comparison of treatment benefit across CDKN3-defined groups, which the current cell-line studies cannot supply.

Prospective studies should specify tumor lineage, etiology, assay and analyte, cutoff, treatment context, and intended use before validation. A candidate marker is more credible when it adds information beyond established clinical and proliferation variables, reproduces in an independent cohort, and corresponds to a measured functional state. There is currently no basis in the cited studies to recommend a selective CDKN3 drug or an expression-based treatment decision.

## Discussion and future directions

Three linked experiments can resolve the principal uncertainties. First, perturb CDKN3 with independent reagents in defined tumor and relevant normal models, then compare rescue with wild-type and catalytically impaired protein. Measure CDK2 Thr160 and CDK1 Thr161 phosphorylation, G1/S and G2/M timing, replication-fork stress, and mitotic progression. The resulting phenotypes should be interpreted alongside enzyme activity and protein localization, rather than inferring activity from transcript levels.

Experimental design should also anticipate competing explanations. CDKN3 depletion can alter the number of cells entering mitosis; if only surviving cells are scored, mitotic abnormalities may appear to rise or fall because of selection. Quantifying protein levels and phosphatase activity after each perturbation, measuring early and late time points, and tracking individual cells would limit that bias. The catalytic-mutant rescue needs comparable expression and localization to the wild-type protein; otherwise a failed rescue could reflect protein instability rather than loss of catalysis. Results should be reproduced in multiple lineages and, for HCC, in models representing distinct etiologies and prior-treatment states.

Second, determine whether altered CDKN3 causes genome instability by quantifying chromosome missegregation, anaphase bridges, micronuclei, karyotype changes, and longitudinal copy-number alterations after matched perturbations. Third, if a CIN phenotype is reproduced, assay cytosolic DNA, cGAS recruitment, phospho-TBK1 and phospho-IRF3, interferon responses, and the consequences of STING knockout or inhibition in immune-competent or otherwise immune-relevant systems. These experiments should treat the CDKN3–CIN–cGAS–STING relationship as a conditional hypothesis rather than a presumed pathway. In immune-relevant cocultures or immunocompetent tumor models, compare secreted interferon, antigen presentation, immune-cell recruitment, and T-cell-mediated killing after CDKN3 perturbation and STING rescue or inhibition.

Limitations. This is a focused narrative review without new experimental data, exhaustive study identification, pooled effect estimates, or formal risk-of-bias grading. Different studies use distinct CDKN3 transcripts, antibodies, etiologies, tissue comparators, cell models, and endpoints; some rely only on computational associations. Direct catalytic measurements and causal tests of the proposed CDKN3–CIN–cGAS–STING connection remain sparse. The evidence tiers in **Table 1** describe what each design can support, not a quantitative score of study quality.

## Conclusions

The CDKN3 paradox is a descriptive mismatch between inhibitory substrate chemistry and frequent associations of high expression with aggressive tumor features. Selected renal models provide perturbation-associated evidence compatible with a tumor-cell contribution; the lung studies cited here primarily provide prognostic and computational association evidence. HCC studies remain directionally discordant across molecular layers and conditions (11–13, 28–35). A context-specific functional dependency would require selective fitness loss after gene-specific perturbation and restoration by an appropriate rescue construct in the tested model; the cited evidence should not be read as establishing this criterion across models. In broader cohorts, CDKN3 expression may primarily reflect proliferation or treatment adaptation. High CDKN3 expression alone does not establish functional dependency, independent prognostic value, treatment prediction, or druggability. CIN and cGAS–STING remain a conditional, testable hypothesis, not an established CDKN3-specific pathway.
