# Supplementary material for: CDKN3 in cancer: context-dependent cell-cycle control and implications for genome stability and therapy
Source: Front Oncol. 2026 Oct 30;16:1991125. doi: 10.3389/fonc.2026.1991125 (PMC13763413; doi:10.3389/fonc.2026.1991125)
Supplement: Supplementary file 1 [file Table1.docx]

Supplementary Table S1. Study-level comparison of 11 hepatocellular carcinoma (HCC) reports illustrating analyte-, etiology-, model-, endpoint-, and treatment-dependent evidence.

Abbreviations: KAP, kinase-associated phosphatase; HCC, hepatocellular carcinoma; HCV, hepatitis C virus; HBV, hepatitis B virus; GEO, Gene Expression Omnibus; TCGA, The Cancer Genome Atlas; LIHC, liver hepatocellular carcinoma; GSE, Gene Expression Omnibus Series; RNA, ribonucleic acid; siRNA, small interfering RNA; LASSO, least absolute shrinkage and selection operator; AKT, AKT serine/threonine kinase; p53, tumor protein p53; p21, cyclin-dependent kinase inhibitor 1A.

| Study | Analyte / layer | Sample and etiology | Model | CDKN3 manipulation | Endpoint | Claim supported | Claim not supported / major limitation |
| --- | --- | --- | --- | --- | --- | --- | --- |
| Yeh et al. (2000) (11) | Aberrant KAP/CDKN3 transcripts; RNA layer | HCC tissue; etiology not specified | Patient specimens; no functional model | None | Transcript identity / aberrant expression | Transcript-level observation of aberrant CDKN3 transcripts. | No inference about protein abundance, phosphatase activity, clinical outcome, or causality. |
| Mori et al. (2022) (12) | CDKN3 expression regulation; RNA / transcriptional layer | HCC expression-regulator study; etiology not specified | HepG2 cell model and expression-regulator assays | USF1/USF2 and CDKN3eRNA-related regulatory analyses | CDKN3 transcript regulation | Identified cell-cycle-associated and -unassociated regulators of CDKN3 expression in the tested system. | Single-cell-line mechanistic scope; does not establish CDKN3-driven tumor growth, patient outcome, or treatment response. |
| Li et al. (2023) (13) | CDKN3 transcript/protein expression in MYC-associated analyses | TCGA-LIHC and MYC-associated HCC models; etiology not fully stratified | Computational analyses with tissue/mouse-model expression validation | No CDKN3-specific perturbation; MYC-associated analyses and validation | Expression, prognosis, and immune/cell-cycle associations | CDKN3 expression was elevated in MYC-high HCC and validated in the examined HCC model/tissues. | MYC-associated expression validation does not isolate CDKN3 causality or establish CDKN3 dependency or clinical prediction. |
| Xing et al. (2012) (28) | CDKN3 overexpression; transcript/protein context | HCC tissues and cell lines; etiology not specified | HCC cell models | Expression comparison with functional suppression as reported | Proliferation and tumor-growth phenotypes | Phenotypic evidence compatible with tumor-supportive activity in the tested models. | No validated rescue, direct activity measurement, or catalytic-dependence evidence. |
| Lin, Lai & Yeh (2013) (29) | KAP/CDKN3 overexpression; RNA/protein context | Alcohol-related HCC | Cell and xenograft models | Antisense suppression | Xenograft growth | Suppression reduced xenograft growth in an alcohol-related HCC model. | Etiology-specific evidence; limited assay, rescue, and target-engagement controls; no clinical target claim. |
| Dai et al. (2016) (30) | Lower CDKN3 protein; protein layer | HCC tissue versus comparator liver; etiology not specified | HCC cell model and patient cohort | siRNA knockdown | Pathological stage, clonogenic survival, p53/p21 and AKT signaling | Protein-level and cellular findings point in a direction different from several overexpression studies. | Comparator tissue, antibody/scoring, and model context may affect direction; no catalytic measurement or wild-type/catalytic-dead rescue. |
| Dai et al. (2020) (31) | Expression–outcome and adriamycin response; treatment-response layer | HCC cohort and in vitro treatment model; etiology not specified | Clinical cohort plus HCC cells | Expression–response analysis; perturbation details limited | Prognosis and adriamycin sensitivity | Prognostic and treatment-response associations were reported in the examined cohort/model. | No treatment-by-marker interaction, prespecified cutoff, catalytic measurement, or clinical predictive evidence. |
| Zhang et al. (2021) (32) | CDKN3 mRNA among hub-gene transcripts; RNA layer | HCV-associated HCC; GEO/TCGA discovery and validation datasets | Bulk transcriptomic and network analyses; no functional model | None; computational association | Differential expression and diagnostic/prognostic associations | CDKN3 was included among HCV-HCC hub genes. | Multigene bulk association does not establish protein/activity, isolated CDKN3 direction, or dependency. |
| Kim et al. (2022) (33) | CDKN3 mRNA in an HCC hub module; RNA layer | HBV-associated HCC; GSE121248 includes 70 HCC liver samples and 37 adjacent-normal liver samples. | Bulk microarray, pathway, and protein-interaction network analysis; no functional model | None; computational association | Tumor-versus-adjacent expression and hub-gene/pathway membership | CDKN3 was identified in an HBV-HCC hub module linked to cell-cycle and division terms. | No CDKN3-specific manipulation, protein/activity measurement, or causal test; sample counts describe the GSE121248 analysis set. |
| Dai et al. (2021) (34) | CDKN3 mRNA in a six-gene risk model; RNA layer | TCGA HCC training cohort; GSE14520 validation; etiology not stratified | GEO/TCGA differential-expression and survival-model analysis; no functional model | None; computational association | Multigene risk score and survival/risk stratification | CDKN3 was included with a negative coefficient in a composite model. | A model coefficient does not establish a protective causal effect or an isolated CDKN3 biomarker. |
| Wu et al. (2022) (35) | CDKN3 mRNA in a nine-gene signature; RNA layer | TCGA-LIHC cohort; etiology not stratified | TCGA-based LASSO Cox risk-signature analysis; no functional model | None; computational association | Overall-survival risk stratification by a multigene signature | CDKN3 was included in a prognostic gene signature. | Signature membership is not gene-specific prognostic validation or functional dependency; no protein/activity or perturbation data. |
